# Emerging Immunotherapies in Lung Cancer: The Latest Advances and the Future of mRNA Vaccines

**DOI:** 10.3390/vaccines13050476

**Published:** 2025-04-28

**Authors:** Raquel Ramos, Nuno Vale

**Affiliations:** 1 PerMed Research Group, RISE-Health, Faculty of Medicine, University of Porto, Alameda Professor Hernâni Monteiro, 4200-319 Porto, Portugal; raquel_ramos00@hotmail.com; 2 RISE-Health, Department of Community Medicine, Health Information and Decision (MEDCIDS), Faculty of Medicine, University of Porto, Rua Doutor Plácido da Costa, 4200-450 Porto, Portugal; 3 Laboratory of Personalized Medicine, Department of Community Medicine, Health Information and Decision (MEDCIDS), Faculty of Medicine, University of Porto, Rua Doutor Plácido da Costa, 4200-450 Porto, Portugal

**Keywords:** lung cancer, therapy, immune system, vaccines, mRNA, mRNA-based vaccines

## Abstract

Lung cancer is the most lethal malignancy worldwide, having the highest incidence rate. This is a heterogeneous disease classified according to its histological and molecular characteristics. Depending on these, different therapeutic approaches have already been approved for lung cancer treatment targeting genetic alterations or even the immune system. Nonetheless, other therapies are being studied to continuously improve the care and survival of lung cancer patients. Among them, immunotherapies are one of the main targets of investigation to try and combat the ability of some malignant cells to evade anti-tumor responses mediated by the immune system. Cancer vaccine development has emerged as a promising approach to strengthen the patient’s immune system and combat the disease, especially mRNA vaccines. Currently, there are several ongoing studies investigating the therapeutic efficacy of mRNA vaccines in lung cancer treatment alone or combined with other therapeutic drugs. This review aims to highlight the importance of immunotherapy in lung cancer treatment, presenting the most recent advances particularly in mRNA-based vaccines as well as the challenges and future perspectives.

## 1. Introduction

Lung cancer (LC) is the most prevalent and lethal cancer worldwide, with approximately 2.5 million new cases and 1.8 million deaths reported in 2022 [1]. Several risk factors play a role in the development of this disease, including tobacco smoking, environmental and occupational exposures, family history of LC, and a history of other lung conditions. Among these, tobacco smoking is a primary risk factor, responsible for about 80% of all LC cases [2]. However, although the exact reasons have not yet been conclusively identified, we are seeing an increase in LC cases in non-smokers, accounting for about 25% of all cases [3,4].

Additionally, LC is known by its poor outcomes once it is diagnosed at advanced stages due to its non-specific symptoms such as persistent cough, chest pain, dyspnea, fatigue, and weight loss [5]. The absence of an established LC screening program also causes this late diagnosis. Currently, the only existing recommendation is annual low-dose computed tomography (LDCT) for high-risk patients—individuals aged 50 to 80 years with a smoking history of at least 20 pack-years [6]. Nonetheless, a European program already exists to help establish LC screening across countries [7]. An early diagnosis is crucial to improve patient survival, enabling earlier therapeutic intervention. However, lung tumors are very heterogeneous, making the therapeutic choice challenging. Therefore, after biopsy, histological tumor classification (small cell lung cancer (SCLC) and non-small cell lung cancer (NSCLC)) and molecular characterization, especially of NSCLC, is crucial for therapeutic decisions because there are targeted therapies that could be applied according to the tumor’s genetic characteristics [8,9]. Recent knowledge on the immune system has also greatly improved cancer immunotherapy [10]. Specifically, in LC patients without driver mutations, treatment with immune checkpoint inhibitors (ICIs), namely PD-L1 and PD-1, is an integrative part of the treatment [11,12,13]. Current treatment recommendations for both SCLC and NSCLC are comprehensively outlined in the clinical guidelines established by leading oncology organizations, including the National Comprehensive Cancer Network (NCCN), American Society of Clinical Oncology (ASCO), and European Society for Medical Oncology (ESMO) [14,15].

Despite the advances, a large number of patients still experience disease progression after immunotherapy, and the efficacy of continuing immunotherapy as second-line treatment in advanced NSCLC and SCLC remains poorly understood [16,17]. Moreover, in the case of SCLC, although combining immunotherapy with platinum-based chemotherapy for extensive stage SCLC has shown benefits, only a small proportion of patients achieved durable responses. Therefore, this highlights the urgent need to identify new biomarkers that can better predict which subgroups of patients with SCLC are most likely to benefit from immunotherapy. Several pre-clinical and clinical trials are actively addressing this challenge [18]. One promising target is the delta-like ligand 3 (DLL3) receptor, which is highly expressed in SCLC and has emerged as a potential focus for immunotherapeutic strategies including bispecific T cell engagers, chimeric antigen receptor T cells (CAR-T cells), and antibody–drug conjugates [19].

### The Importance of the Immune System

Currently, it is known that cancer progression hardly depends on the interaction between cancer cells and their microenvironment, particularly its immunological components [20]. Several components of the immune system are responsible for the immunosurveillance against tumors. Specifically, the immune system can be divided into two main responses: adaptive and innate. Both play important roles in protecting the body against tumors by working together to recognize and eliminate cancer cells [20,21].

Specifically, the innate immune system is essential for the immediate detection and subsequent elimination of cancer cells. On the other hand, the adaptive immune system depends on antigen-presenting cells, like dendritic cells (DCs), to activate T cells. This activation allows T cells to recognize antigenic epitopes (tumor-specific markers) that appear at the beginning and during tumor progression [20,22,23,24].

However, despite the complexity of immune system mechanisms, cancer cells can evade the immune system through various strategies including the downregulation of surface antigens to reduce visibility to immune cells, the secretion of immunosuppressive factors such as TGF-β, IL-10, CXCLs, and the expression of immune checkpoint molecules like PD-L1 and CTLA-4, which bind to T cells and inhibit their activity [25]. As previously mentioned, in LC, immunotherapy using ICIs is already an approved therapeutic approach. Nonetheless, apart from ICIs, new technical advances have helped in better knowledge regarding LC immunogenicity, leading to an evolution of various types of immunotherapies for LC treatment (Table 1) [10]. Therapeutic vaccines, as outlined in the next table, are also emerging as a promising option in LC treatment. Oncolytic virus therapy is another option being studied in clinical trials due to its ability to target, infect, and destroy tumor cells within the tumor microenvironment [26]. Additionally, chimeric antigen receptor (CAR) T-cell therapy shows potential as it involves genetically modifying T cells to express a CAR with an intracellular signaling domain and an extracellular antigen-recognition structure, specifically targeting tumor-associated antigens (TAAs) [27]. In NSCLC, immunotherapy is no longer limited to the advanced stage and has also gained FDA approval for use in earlier stages, including perioperative and adjuvant treatment settings. Multiple clinical trials have demonstrated its efficacy when used as a neoadjuvant therapy, offering several benefits such as the earlier targeting of micro-metastatic disease, stronger immune activation, better pathological response rates, and fewer delays in treatment. Nonetheless, as with SCLC, there is a growing emphasis on the identification of predictive biomarkers to guide patient selection, allowing for more personalized and effective immunotherapy strategies in NSCLC [28].

Considering the importance of the immune system in LC treatment and the growing research in this area, this article provides an in-depth exploration of therapeutic cancer vaccines, with particular emphasis on their application in LC treatment. We examine the current status of the vaccine-based immunotherapy field, highlighting advancements and clinical developments. Special attention is given to the development and progress of mRNA vaccines, a rapidly evolving and promising strategy in LC treatment. The advantages and the main problems and challenges of mRNA vaccines are also discussed in the article. By reviewing these points, we aim to offer a comprehensive overview of how therapeutic vaccines, especially mRNA-based vaccines, are influencing the future of LC therapy.

## 2. The Beginning of Vaccines and the Transition to Cancer

**Figure 1:**
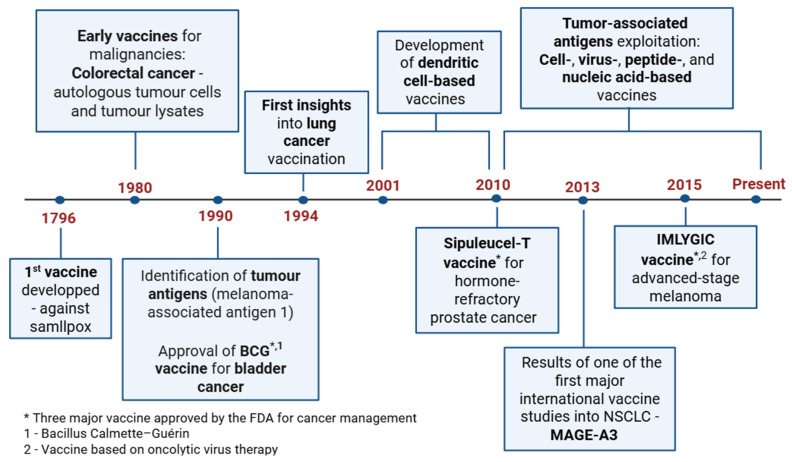
Evolution of vaccines from their first development up to their application in cancer treatment. Created with BioRender.com. Available online: http://biorender.com/ (accessed on 8 April 2025).

In 1796, the first vaccine was created, being against smallpox, and the development of this technology grew exponentially in the subsequent centuries [34]. Over the years, progress has been made in this area with the discovery of biological factors useful for vaccine development, such as autologous tumor cells, tumor lysates, and tumor antigens. Consequently, due to all of these findings, the creation of cancer vaccines has become a reality [35,36,37,38,39,40,41]. Figure 1 represents the timeline of therapeutic vaccine development from its creation up to its research and application in cancer.

As stated in Figure 1, tumor-associated antigen (TAA) vaccines are the most recently studied and comprise four major types: cell-based vaccines; virus-based vaccines; peptide-based vaccines; and nucleic-acid based vaccines (DNA and RNA) [39,42].

### Vaccines for Cancer Treatment

Cancer vaccines are an alternative immunotherapeutic approach with both therapeutic and prophylactic (HPV and hepatitis B vaccines) potential. Like other cancer immunotherapies, cancer vaccines also aim to slow tumor progression and enhance the patients’ survival by stimulating the host anti-tumor immunity and adapting the suppressive tumor microenvironment in a more targeted, safe, and tolerable manner. The TAAs hold particular promise since they can induce a long-lasting therapeutic effect due to immunologic memory. However, translating cancer vaccines into effective clinical therapies is challenging due to highly variable tumor antigens and a relatively low immune response. Consequently, several vaccine candidates and personalized cancer vaccines combined with checkpoint inhibitors or cytokine therapies are under investigation in both pre-clinical and clinical stages to improve their efficacy and enable their clinical application [39,43]. Among the four previously defined vaccine types, nucleic acid-based vaccines have emerged as one of the most promising approaches. First of all, this type of vaccine offers the advantage of delivering multiple antigens at once, targeting various TAAs or somatic mutations, enhancing both humoral and cell-mediated immune responses, and reducing the possibilities of resistance. Moreover, contrary to peptide vaccines, they are less dependent on specific human leukocyte antigen (HLA), once nucleic acid vaccines encode full-length tumor antigens, increasing the chances of inducing a stronger and more diverse immunity response by T cells. Finally, their non-infectious nature, liberty from protein or virus-derived contaminations, and tolerance for both prophylactic and therapeutic applications, with a relatively low production cost and ease of manufacturing, are other important advantages of acid nucleic vaccines [39,44,45,46].

#### Vaccines for LC Treatment

As stated at the beginning, the immune system plays an important role in cancer treatment. In LC, the exploration of alternative therapeutic strategies using the immune system is increasing due to immunotherapy’s safety profile, effectiveness, and long-lasting therapeutic response [47]. Across these decades, efforts have been made to create vaccines for LC, and, recently, radiotherapy has been linked to immunotherapy, showing that it may enhance the anti-tumor immunity of cancer cell vaccines. Concomitantly, the combination of immunotherapy with conventional therapies, such as chemotherapy, has also been demonstrated to enhance apoptosis, provide long-lasting responses, and decrease the secondary effects [37,48].

Specifically in NSCLC, the use of ICIs has become the standard choice in patients without driver mutations, however, the survival rate remains poor [49,50,51,52]. Consequently, due to the immunosuppressive tumor microenvironment and the limited responses to ICIs, cancer vaccines have emerged as a promising approach to improve NSCLC patient survival. In addition, NCSLC has arisen as a prime candidate for cancer vaccine development due to its high genetic heterogeneity, allowing for the development of personalized cancer vaccines and improving the precision, safety, and effectiveness of therapy [53]. Table 2 shows different therapeutic vaccines that have been developed for NSCLC treatment.

## 3. mRNA Vaccines

Despite all the advantages of the acid nucleic vaccines described, RNA-based vaccines have arisen as an interesting alternative to DNA vaccines for cancer treatment, especially vaccines using messenger RNA (mRNA) [39,44], and its popularity has risen with COVID-19 vaccines [39,54,55,56]. This type of vaccine triggers an immune response by targeting specific tumor antigens. These antigens are generally categorized into two types: TAAs, which are normally found in the body but are overexpressed in cancer cells; and tumor neoantigens, which are the result of genetic mutations exclusive to tumor cells and are detected by antigen-specific T cell receptors (TCRs) in conjunction with major histocompatibility complex (MHC) molecules. In practice, most developed mRNA vaccines encode one of the two types of tumor antigens, with common examples including carcinoembryonic antigen (CEA), prostate-specific antigen (PSA), melanoma-associated antigen (MAGE) 1, survivin, tyrosinase, human telomerase reverse transcriptase (hTERT), and Wilms’ tumor 1 antigen (WT1) [57,58].

Compared with DNA vaccines, mRNA vaccines have multiple advantages, namely being the simplest nucleic acid vaccines, having a higher immunogenicity, cannot integrate into the genome sequence, are free of insertional mutagenesis, and RNA simply needs to enter the cytoplasm, where it undergoes a single-step translation to produce the desired antigen [39,42,59]. Furthermore, mRNA’s ability to encode multiple antigens at once enhances the overall effectiveness of the vaccine and supports the creation of personalized therapies. The temporary nature of mRNA expression also limits prolonged antigen stimulation, reducing the likelihood of chronic inflammation or autoimmune responses [60]. However, mRNA vaccines also have some disadvantages, namely RNA instability and quick degradation by extracellular RNases, insufficient in vivo delivery, and high intrinsic innate immunogenicity [61]. Thus, over the years, efforts have been made to improve mRNA stability and its in vivo delivery. DCs were the main platforms used for mRNA vaccines in older studies. However, more recently, other delivery systems, such as the formulation of mRNA into delivery vehicles including lipid nanoparticles (LNPs), polymers, and peptides, have been developed. Nevertheless, most mRNA vaccine approaches use lipid nanoparticulate formulation carriers [39,62,63]. A key feature of LNP-based mRNA vaccines is their role as adjuvants, acting as powerful immunostimulatory agents that enhance both the quality and magnitude of adaptive immune responses. This strong immune activation induced by mRNA-LNPs has already been demonstrated in multiple pre-clinical and clinical trials. Recent research further supports the idea that LNPs boost immune responses, showing that they significantly enhance T follicular helper cell activity and humoral immunity [64,65]. Moreover, studies have shown that empty LNPs can elicit a therapeutic response by promoting antibody production, triggering the release of chemokines, and promoting the infiltration of neutrophils and monocytes in the injection site [66]. Another study also demonstrated that the amine groups present in the lipid compounds of the particles interacted with immune cells, activating both an innate and adaptive immune system response, and increasing the pro-inflammatory cytokines. Nonetheless, this interaction can also lead to the release of compounds that may reduce the efficacy of mRNA-LNP [67]. Therefore, while all of these findings reinforce that LNPs not only serve as delivery agents for mRNA vaccines but also act as potent immunostimulatory agents, their design must ensure favorable immunological profiles for optimal efficacy.

Moreover, another important characteristic of mRNA-LNP vaccines is their ability to induce a robust immune response MHC-I presentation as they can express intracellular antigens that are processed and presented on the surface of antigen-presenting cells (APCs). This MHC-I–peptide complex is recognized by CD8+ T cells, which can then target and eliminate tumor cells [68].

**Figure 2:**
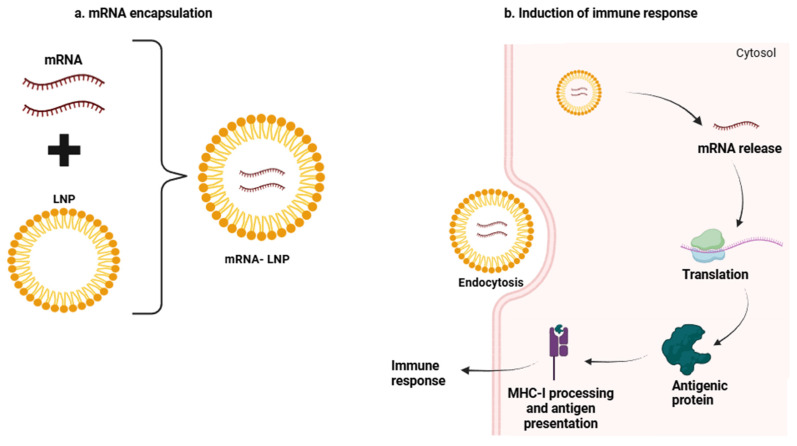
mRNA vaccine formulation and immune response induction. (**a**) mRNA is encapsulated within a lipid nanoparticle (LNP) to enhance stability and facilitate efficient delivery. (**b**) The mRNA–LNP complex enters the cell through endocytosis, where the mRNA is released into the cytosol and translated into an antigenic protein. This protein binds to the major histocompatibility complex I (MHC-I), being presented to cytotoxic T cells, activating them, and finally triggering an immune response. Created with BioRender.com. Available online: http://biorender.com/ (accessed on 12 February 2025).

Figure 2 represents the mechanism of mRNA vaccines using LNP technology.

Due to the clinical potential of mRNA vaccines as previously described, several Phase I and II clinical trials were and are being performed in various cancer types such as ovarian cancer, triple-negative breast cancer, prostate cancer, melanoma, and lung cancer [62,69]. Table 3 shows the ongoing Phase I and II clinical trials in several types of cancers.

## 4. mRNA Vaccines for LC

Among all of the LC vaccines under study presented above, mRNA vaccines have had promising results, overcoming the main problems of mRNA vaccines previously described [70].

Over the years, several studies have been conducted to assess the safety and effectiveness of different mRNA vaccines as monotherapy or in combination with other regimens [71]. In the beginning, DC-based vaccines containing mRNA encoding CEA were evaluated in a Phase I clinical trial involving patients with metastatic cancers that expressed CEA, including lung cancer. The results showed that this vaccine was safe without major toxicities observed [72].

Despite the previous efforts to develop mRNA vaccines for lung cancer treatment and improve their therapeutic efficacy, new approaches have emerged that aim to further enhance their effectiveness, particularly through the use of LNP formulations, the inclusion of multiple lung tumor-associated antigens, or in combination with other immunotherapies. A recent Phase I clinical trial (NCT03948763) assessed an LNP-formulated mRNA vaccine (mRNA-5671/V941), both as an independent treatment and in combination with pembrolizumab, in patients with KRAS-mutant NSCLC and other solid tumors to identify the recommended dose. The study has already finished; however, no results have been published. CV9201 is another mRNA-based cancer vaccine developed for the treatment of stage IIIB/IV NSCLC. This vaccine has mRNAs encoding five NSCLC-related antigens (New York esophageal squamous cell carcinoma-1 (NY-ESO-1), melanoma antigen family C1 and C2, survivin, and trophoblast glycoprotein (5T4)) and was tested in a Phase I/II clinical trial. The results indicated that CV9201 was well-tolerated with the recommended dose for Phase IIa set at 1600 µg, and although immune activation was infrequent, there was some evidence of it [73]. Additionally, a successor vaccine—CV9202—was investigated in combination with local radiation therapy in patients with stage IV NSCLC (NCT01915524), encoding six NSCLC-associated antigens: Mucin1 (MUC1), survivin, NY-ESO-1, 5T4, MAGE-C2, and MAGE-C1. The treatment was well-tolerated, and antigen-specific immune responses were also detected [74]. These promising results encourage the further investigation of CV9202 in combination with ICIs, and a Phase I/II study (NCT03164772) has already determined the safety and efficacy of CV9202 plus the anti-PD-L1 antibody durvalumab or CV9202 plus durvalumab plus the CTLA-4 antibody tremelimumab. The results showed that patients treated with both ICIs had a higher rate of disease progression (59.3%) compared with those treated with the mRNA vaccine plus durvalumab (36.8%). On the other hand, the mRNA vaccine plus durvalumab group had a better partial response (26.3% vs. 11.1%) [71,75].

Currently, another mRNA vaccine—BNT116—is under investigation in combination with the PD-1 inhibitor cemiplimab for patients with advanced NSCLC (NCT05142189 and NCT05557591) [76,77]. In complement, the NCT03908671 trial is studying a personalized neoantigen mRNA vaccine to assess its safety and tolerability.

Beyond clinical studies, researchers are actively exploring new LC antigens for mRNA vaccine development. A recent study on lung adenocarcinoma (LUAD) identified several potential antigens and categorized LUAD patients into three ferroptosis subtypes (FS1, FS2, and FS3), each with distinct immune checkpoints and immunogenic cell death modulators. FS3, which exhibited the highest tumor mutation burden, emerged as the most promising candidate for mRNA vaccine effectiveness. The study highlighted six antigens—AGPS, NRAS, MTDH, PANX1, NOX4, and PPARD—as particularly suitable targets for mRNA vaccination, especially in FS3 tumors [78].

Although all investigations presented showed promising results with several positive outcomes, it is important to emphasize that certain limitations must be considered. Patient variability is a key factor; each patient has their own biologic and physical characteristics that can influence the vaccine’s therapeutic effect, reducing or exacerbating the immune response. Additionally, another important point that must be carefully monitored is the immune-related adverse events that may arise from vaccines, including both immediate and delayed side effects. Moreover, the long-term effects of the vaccines under testing also require a thorough evaluation in order to determine their duration of therapeutic action, and also how often booster doses may be needed to maintain their effect. Therefore, the described studies need to proceed with this kind of research to fully assess the real efficacy and safety of mRNA vaccines in the treatment of LC.

Therefore, developing cancer vaccines is challenging due to the weak immunogenic character and high variability of TAAs across patients, the difficulty in efficiently achieving both humoral and cellular immunity, the suppressive microenvironment created by the tumor, and the complexity of identifying and delivering highly immunogenic tumor-specific antigens. The need for repeated high-dose administrations also raises safety concerns. Additionally, overcoming immune evasion mechanisms and optimizing vaccine formulations to ensure long-lasting and effective responses remain critical obstacles in cancer vaccine development. Therefore, future investigations are needed to improve the efficacy of mRNA vaccines and enhance their translation into clinical practice [39]. One potential approach may involve exploring the lung microbiome, which is increasingly recognized for its influence on immunotherapy outcomes. A novel strategy could include designing mRNA vaccines that encode molecules capable of modulating the lung microbiome, and consequently, improve the anti-tumor responses. Another important direction lies in the field of personalized medicine addressing tumor heterogeneity. This approach could focus on developing individualized mRNA vaccines that encode patient-specific neoantigens. Moreover, targeting the tumor microenvironment is a valuable strategy. Developing new mRNA vaccines coding proteins capable of modulating immunosuppressive factors within the tumor could help restore immune activity and improve therapeutic efficacy. Since we are in the technology and artificial intelligence era, it would be interesting to develop machine learning tools to predict the most immunogenic factors and neoantigens shared across populations, which could significantly accelerate vaccine design and optimization. Finally, although more technically demanding, the intratumoral administration of mRNA vaccines offers a promising route. Delivering the vaccine directly into the tumor site could stimulate local antigen-presenting cells and promote immune cell infiltration, potentially enhancing the anti-tumor immune response.

## 5. Conclusions

Lung cancer remains the leading cause of cancer-related deaths worldwide, and despite advances in therapeutic approaches, the overall survival rates continue to be poor. Over the years, the role of the immune system in lung cancer treatment has gained importance, however, some issues such as therapeutic resistance and tumor progression persist in both SCLC and NSCLC. Consequently, several research trials are focused on investigating predictive biomarkers that can optimize the use of immunotherapy in lung cancer. Within this evolving landscape, therapeutic vaccine has emerged as a promising option to enhance patient outcomes. More specifically, mRNA vaccines are gaining attention as therapeutic alternatives due to their high immunogenic potential, simplified manufacturing processes, and capacity for personalization. Numerous clinical trials are currently investigating mRNA-based vaccines, particularly for NSCLC, with encouraging preliminary results. Interestingly, radiation therapy has been shown to enhance the immune response triggered by mRNA vaccines. As a result, some studies are investigating the therapeutic effects of mRNA vaccines both as a monotherapy and in combination with radiotherapy or ICIs, yielding promising therapeutic benefits. Nonetheless, to fully harness the potential of mRNA vaccines, more comprehensive studies are needed. Problems such as interpatient variability, immune-related adverse events, and long-term efficacy of the vaccines must be carefully addressed. Concomitantly, despite the LNP delivery system having several advantages, being one of the most widely used systems, the development of mRNA vaccines is still challenging, and further studies are needed to improve the efficacy of these treatment approaches. Future research should focus on enhancing vaccine delivery and immune response through exploration of the tumor microenvironment, lung microbiome modulation, and intratumoral administration. Personalized medicine, supported by artificial intelligence tools for identifying shared immunogenic targets, may also play a key role in refining these therapeutic strategies and expanding their clinical success.

## Figures and Tables

**Table 1:** Therapies targeting the immune system for lung cancer treatment.

Immunotherapy Type	Description	Refs.
Immune checkpoint inhibitors (ICIs)	CTLA-4, PD-1, and PD-L1 inhibitors represent the most successful immunotherapeutic strategy in NSCLC	[12]
Oncolytic virus therapy (Ovs)	Genetically-modified viruses selectively target and destroy cancer cells, triggering an immune response and inhibiting tumor progression	[26]
CAR-T	Genetically-engineered autologous or allogeneic T cells are modified in vitro to express specific T-cell receptors that recognize cancer antigens	[27,29]
Bispecific T-cell engagers [BiTEs]	Antibody-based therapy that connects the patient’s own T cells to cancer cells, activating the T cells’ cytotoxic function without the need for genetic modification or external manipulation of the T cells	[30]
Therapeutic vaccines	Enhance adaptive anti-tumor immune responses by introducing tumor-specific antigens to activate the host immune system	[31,32,33]

**Table 2:** Different possibilities of therapeutic cancer vaccines for NSCLC. Identification by the ClinicalTrials.gov Identifier is represented [53].

Vaccine Type	Formulation	Trial Phase	Trail Identification and Target
Cancer cell vaccine	Inactivated tumor cells or lysated	Clinical trial	NCT00676507—Stages III or IV of NSCLC
Dendritic cell vaccine	Protein/peptide; DNA; RNA; chemokines	Clinical trial	NCT03546361—Stage IV of NSCLC
Protein/peptide vaccine	Recombinant protein; peptide	Clinical trial	NCT04298606—preventing NSCLC recurrence in IB–IIIA stages (production of antibodies against EGF)
DNA vaccine	DNA	Clinical trial	NCT05242965—Stage IV of NSCL (given with GM-CSF to help create a stronger immune response
mRNA vaccine	mRNA	Clinical trial	NCT05557591—Advanced NSCLC with tumors expressing PD-L1 ≥ 50%
circRNA ^1^ vaccine	circRNA	Preclinical trial	-
Neoantigen vaccine	Peptide; protein; mRNA	Clinical trial	NCT03948763—Advanced or metastatic NSCLC with KRAS mutant

^1^ Circular RNA.

**Table 3:** Ongoing mRNA vaccine Phase I and Phase II clinical trials for different cancer types. Identification by the ClinicalTrials.gov Identifier is represented [62,69].

Cancer Type	Trial Phase	Combination	CLINICALTRIALS.GOV Identifier
Solid tumors	Phase I/Phase II	Chimeric antigen receptor therapy	NCT04503278
Solid tumors	Phase I	Atezolizumab	NCT03289962
Resected solid tumors	Phase I	Pembrolizumab	NCT03313778
Melanoma	Phase II	Pembrolizumab	NCT03897881
Melanoma	Phase II	Cemiplimab	NCT04526899
NSCLC ^1^	Phase I	Cemiplimab + docetaxel	NCT05142189
Pancreatic cancer	Phase I	Oxaliplatin, irinotecan, fluorouracil, leucovorin, and atezolizumab	NCT04161755
Head and neck squamous cell carcinoma	Phase II	Pembrolizumab	NCT04534205
CRC ^2^	Phase II	None	NCT04486378
Glioblastoma	Phase I	None	NCT04573140

^1^ Non-small cell lung cancer; ^2^ Colorectal cancer.

## Data Availability

Not applicable.

## Abbreviations

The following abbreviations are used in this manuscript:

APCs	Antigen-presenting cells
DCs	Dendritic cells
ICIs	Immune checkpoint inhibitors
LC	Lung cancer
LUAD	Lung adenocarcinoma
NSCLC	Non-small cell lung cancer
SCLC	Small cell lung cancer
TAAs	Tumor-associated antigens
